# RETRACTION: Impact of drying methods on composition and functional properties of date powder procured from different cultivars

**DOI:** 10.1002/fsn3.4090

**Published:** 2024-04-05

## Abstract

**RETRACTION:** Nighat Raza, Muhammad U. Arshad, Faqir M. Anjum, Farhan Saeed, Abid A. Maan, and Huma Bader Ul Ain, “Impact of drying methods on composition and functional properties of date powder procured from different cultivars.” *Food Science & Nutrition* 7 (2019): 2345–2352, https://doi.org/10.1002/fsn3.1081.

The above article, published online on 04 June 2019 in Wiley Online Library (wileyonlinelibrary.com), has been retracted by agreement between the journal's Editor‐in‐Chief, Y. Martin Lo, and Wiley Periodicals, LLC. The retraction has been agreed to following concerns about the peer review process. The editorial office found unambiguous evidence that the manuscript was accepted solely based on insufficient peer review reports. This evidence was further investigated and verified by the publisher and the Editor‐in‐Chief. As a result, the conclusions reported in the article are not considered reliable as the article was accepted without peer review. The authors were notified of the retraction and disagreed with this decision.

**RETRACTION:** Nighat Raza, Muhammad U. Arshad, Faqir M. Anjum, Farhan Saeed, Abid A. Maan, and Huma Bader Ul Ain, “Impact of drying methods on composition and functional properties of date powder procured from different cultivars.” *Food Science & Nutrition* 7 (2019): 2345–2352, https://doi.org/10.1002/fsn3.1081.

The above article, published online on 04 June 2019 in Wiley Online Library (wileyonlinelibrary.com), has been retracted by agreement between the journal's Editor‐in‐Chief, Y. Martin Lo, and Wiley Periodicals, LLC. The retraction has been agreed to following concerns about the peer review process. The editorial office found unambiguous evidence that the manuscript was accepted solely based on insufficient peer review reports. This evidence was further investigated and verified by the publisher and the Editor‐in‐Chief. As a result, the conclusions reported in the article are not considered reliable as the article was accepted without peer review. The authors were notified of the retraction and disagreed with this decision.

